# Prognostic Impact of Blood Lipid Profile in Patients With Advanced Solid Tumors Treated With Immune Checkpoint Inhibitors: A Multicenter Cohort Study

**DOI:** 10.1093/oncolo/oyad273

**Published:** 2023-10-05

**Authors:** Federica Pecci, Luca Cantini, Valeria Cognigni, Fabiana Perrone, Giulia Mazzaschi, Veronica Agostinelli, Giulia Mentrasti, Elda Favari, Michele Maffezzoli, Alessio Cortellini, Francesca Rossi, Rebecca Chiariotti, Francesco Maria Venanzi, Giuseppe Lo Russo, Giulia Galli, Claudia Proto, Monica Ganzinelli, Francesca Tronconi, Francesca Morgese, Carla Campolucci, Marco Moretti, Arianna Vignini, Marcello Tiseo, Roberta Minari, Marco Luigi Bruno Rocchi, Sebastiano Buti, Rossana Berardi

**Affiliations:** Department of Medical Oncology, Università Politecnica delle Marche, AOU delle Marche, Ancona, Italy; Department of Medical Oncology, Università Politecnica delle Marche, AOU delle Marche, Ancona, Italy; Department of Pulmonary Medicine, Erasmus MC Cancer Institute, University Medical Center, Rotterdam, The Netherlands; Fortrea, Inc., Durham, NC, USA; Department of Medical Oncology, Università Politecnica delle Marche, AOU delle Marche, Ancona, Italy; Department of Medicine and Surgery, University of Parma, Parma, Italy; Medical Oncology Unit, University Hospital of Parma, Parma, Italy; Department of Medicine and Surgery, University of Parma, Parma, Italy; Medical Oncology Unit, University Hospital of Parma, Parma, Italy; Department of Medical Oncology, Università Politecnica delle Marche, AOU delle Marche, Ancona, Italy; Department of Medical Oncology, Università Politecnica delle Marche, AOU delle Marche, Ancona, Italy; Department of Food and Drug, University of Parma, Parma, Italy; Department of Medicine and Surgery, University of Parma, Parma, Italy; Medical Oncology Unit, University Hospital of Parma, Parma, Italy; Division of Cancer, Department of Surgery and Cancer, Imperial College London, Hammersmith Hospital, London, UK; Department of Medical Oncology, Università Politecnica delle Marche, AOU delle Marche, Ancona, Italy; Department of Medical Oncology, Università Politecnica delle Marche, AOU delle Marche, Ancona, Italy; Department of Medical Oncology, Università Politecnica delle Marche, AOU delle Marche, Ancona, Italy; Department of Medical Oncology, Fondazione IRCCS Istituto Nazionale dei Tumori, Milan, Italy; Department of Medical Oncology, Fondazione IRCCS Istituto Nazionale dei Tumori, Milan, Italy; Department of Medical Oncology, Fondazione IRCCS Istituto Nazionale dei Tumori, Milan, Italy; Department of Medical Oncology, Fondazione IRCCS Istituto Nazionale dei Tumori, Milan, Italy; Department of Medical Oncology, Università Politecnica delle Marche, AOU delle Marche, Ancona, Italy; Department of Medical Oncology, Università Politecnica delle Marche, AOU delle Marche, Ancona, Italy; SOD Medicina di Laboratorio, Azienda Ospedaliera Universitaria delle Marche, Ancona, Italy; SOD Medicina di Laboratorio, Azienda Ospedaliera Universitaria delle Marche, Ancona, Italy; Department of Clinical Sciences, Università Politecnica delle Marche, Ancona, Italy; Department of Medicine and Surgery, University of Parma, Parma, Italy; Medical Oncology Unit, University Hospital of Parma, Parma, Italy; Department of Medicine and Surgery, University of Parma, Parma, Italy; Medical Oncology Unit, University Hospital of Parma, Parma, Italy; Biomolecular Sciences Department, University of Urbino, Urbino, Italy; Department of Medicine and Surgery, University of Parma, Parma, Italy; Medical Oncology Unit, University Hospital of Parma, Parma, Italy; Department of Medical Oncology, Università Politecnica delle Marche, AOU delle Marche, Ancona, Italy

**Keywords:** lipid metabolism, immune checkpoint inhibitors, lipid profile, triglycerides-high-density lipoproteins ratio

## Abstract

**Background:**

Specific components of lipid profile seem to differently impact on immune activity against cancer and unraveling their prognostic role in patients with solid cancer treated with immune checkpoint inhibitors (ICIs) is needed.

**Materials and Methods:**

We retrospectively collected baseline clinicopathological characteristics including circulating lipid profile (total cholesterol [TC], triglycerides [TG], low-density lipoproteins [LDL], high-density lipoproteins [HDL]) of patients with consecutive solid cancer treated with ICIs, and we investigated their role in predicting clinical outcomes.

**Results:**

At a median follow-up of 32.9 months, among 430 enrolled patients, those with TC ≥ 200 mg/dl showed longer median progression-free survival (mPFS; 6.6 vs. 4.7 months, *P* = .4), although not reaching statistical significance, and significantly longer median overall survival (mOS; 19.4 vs. 10.8 months, *P* = .02) compared to those with TC < 200 mg/dl. Conversely, patients with TG ≥150 mg/dl displayed shorter PFS (3.4 vs. 5.1 months, *P* = .02) and OS (7.1 vs. 12.9 months, *P* = .009) compared to those with TG <150 mg/dl. TC and TG were then combined in a “LIPID score” identifying three subgroups: good-risk (GR) (TC ≥200 mg/dl and TG <150 mg/dl), intermediate-risk (IR) (TC <200 mg/dl and TG <150 mg/dl or TC ≥200 mg/dl and TG ≥150 mg/dl) and poor-risk (PR) (TC <200 mg/dl and TG ≥150 mg/dl). The mPFS of GR, IR, and PR groups was 7.8, 4.3, and 2.5 months, respectively (*P* = .005); mOS of GR, IR, and PR was 20.4, 12.4, and 5.3 months, respectively (*P* < .001). At multivariable analysis, the PR profile represented an independent poor prognostic factor for both PFS and OS.

**Conclusions:**

We developed a lipid score that defined subgroups of patients with cancer who differently benefit from ICIs. Further mechanistic insights are warranted to clarify the prognostic and predictive role of lipid profile components in patients treated with ICIs.

## Teaser

Specific components of lipid profile have different effects on immune activity against cancer. This study aimed to unravel their prognostic role in solid tumors treated with immune checkpoint inhibitors.

**Implications for Practice**

In patients with advanced solid tumors treated with immune checkpoint inhibitors, combining total cholesterol with triglycerides in a “lipid score” allowed us to define three subgroups of patients with different survival benefit from immune checkpoint inhibitors. Among total cholesterol components, HDL, but not LDL, had an impact on patient survival, and combining HDL with triglycerides, we were able to define again 3 subgroups of patients with different survival benefit. The assessment of baseline patient lipid profile before immune checkpoint inhibitors therapy may represent a useful and easily available tool to guide clinical-decision making and stratify prognosis of patients’ with cancer.

## Introduction

In the last decade, immune checkpoints inhibitors (ICIs) have led to a significant survival benefit across different cancer types. However, a considerable proportion of patients with cancer still do not benefit from ICIs because of innate and acquired resistance.^1^ Therefore, identification of prognostic or predictive factors for patients treated with ICIs represents a field of active research. Cholesterol and other components of lipid profile have been assessed as determinants of several alterations occurring in immune cells.^2-4^ In mice models, hypercholesterolemia led to increased cholesterol accumulation into NK cells, increased lipid raft formation, and immune signaling activation.^5,6^ Cholesterol accumulation on the cell membrane of monocyte-derived dendritic cells (moDCs) was shown to enhance major histocompatibility complex (MHC) II-dependent antigen presentation and CD4+ T-cell activation.^7^ By associating with the T-cell receptor (TCR) β chain, cholesterol may be also able to increase TCR nanoclustering and signaling, leading to more efficient formation of immunological synapses on CD8+ T cells.^8^ Drugs used to regulate the lipid metabolism, such as statins, may also play a role, as they enhance antigen presentation and immunogenicity of tumor cells, by inhibiting protein prenylation through the mevalonate pathway and increasing expression of MHC class I on tumor membrane.^9-11^ Unbalanced lipid profile is commonly associated with diabetes and cardiovascular diseases, which might impact on overall survival of patients with cancer. Therefore, while lipid profile components seem to modulate anti-tumor immune response, their association with other comorbidities might additionally contribute to patient prognosis under ICIs treatment.^12-14^

To date, components of circulating lipid profile have been separately investigated in the setting of patients with cancer treated with ICIs and integrating them in a lipid signature might improve patient stratification. Aim of this study is to understand the impact of distinct circulating components of lipid profile on outcomes of patients with advanced solid cancer undergoing ICIs and provide a blood lipid signature able to identify patients more likely to benefit from ICI treatment.

## Material and Methods

### Study Design and Study Population

We retrospectively collected and analyzed clinicopathological data from patients diagnosed with advanced solid tumors including non-small cell lung cancer (NSCLC), melanoma, renal cell carcinoma (RCC), head and neck carcinoma, urothelial carcinoma, small cell lung cancer, and breast cancer. Patients were included if treated with ICIs, alone or in combination with tyrosine kinase inhibitors (TKI) or chemotherapy schedules according to approved oncological indication between January 2016 and December 2021. Patients were identified from patient electronic records of the Polytechnic University of Marche (Ancona), National Cancer Institute (INT, Milan), and University Hospital of Parma (Parma). Only patients with available plasmatic lipid profile, either complete or partial (total cholesterol availability was mandatory for inclusion in the study), collected no earlier than 45 days before starting ICIs were included in the analysis. Baseline circulating lipid profile included total cholesterol (TC), triglycerides (TG), low-density lipoproteins (LDL), high-density lipoproteins (HDL). TC, TG, LDL, and HDL cutoffs for normality, according to American Heart Association, were ≥ 200 mg/dl for TC, ≥ 150 mg/dl for TG, ≥ 100 mg/dl for LDL, < 40 mg/dl for HDL in males, and < 50 mg/dl for HDL in females.^15^ To further investigate the patient metabolic profile at the time of ICIs start, we reviewed patient medical history for cardiovascular (CV) events, defined as any type of disease that affects the heart or blood vessels according to National cancer Institute and American Heart Association definitions,^16,17^ diabetes mellitus (DM), hypertension (HT), statin use at baseline, and body mass index (BMI). BMI was calculated using the formula of weight/height^2^ (kilogram/square meter). Patients with a BMI between 25 and 29.9 kg/m^2^ (overweight) and ≥ 30 BMI kg/m^2^ (obese) were compared to patients with a BMI < 25 kg/m^2^, that included patients with normal weight (BMI: 18.5-24.9 kg/m^2^) and underweight patients (BMI < 18.5 kg/m^2^), according to the WHO categories. To avoid the negative prognostic impact of cachexia, we performed a second analysis comparing patients with BMI ≥ 25 kg/m^2^ and patients with normal weight, as previous reported.^18^

Response to ICIs was evaluated according to RECIST criteria (version 1.1).^19^ Disease control rate (DCR) was defined as the proportion of patients with radiological evidence of complete response, partial response, and stable disease. Progression-free survival (PFS) and overall survival (OS) were calculated from the time of ICI initiation (as monotherapy or in combination) until radiological progression or death/last follow-up for PFS and until death/last follow-up for OS. For patients who did not progress, censoring was established at the time of last radiological evaluation without evidence of progression; patients still alive at the time of data analysis were censored considering the time of last contact. Ethical approval to conduct this study was obtained by the respective local ethical committees on human experimentation of each participating center, after previous approval by the coordinating center (“Comitato Etico Regionale delle Marche - C.E.R.M.,” Reference Number 19/792). All study-related procedures and data collection were conducted in accordance with the Declaration of Helsinki and in accordance with Good Clinical Practice.

### Statistical Analysis

Demographic, clinicopathological, and treatment data were abstracted from electronic medical records. Baseline characteristics were presented using count and percentage for categorical variables, median, and range for continuous variables. To compare proportions across groups, Pearson chi-square or Fisher’s exact tests were used for categorical variables and Mann-Whitney *U* test or the Kruskal-Wallis test for continuous variables. Survival curves were plotted using the Kaplan-Meier (KM) method and differences in probability of surviving between the strata were evaluated by log-rank (Mantel-Cox) test. Median follow-up was calculated using reverse KM method. The hazard ratios (HR) of progression and death were calculated using univariable/multivariable Cox proportional hazard model. Besides the lipid profile, the following covariates were included in the univariable model: age (< 70 vs. ≥ 70 years old), sex (female versus male), tumor type (NSCLC vs Others), lines of treatment (first vs. ≥ 2), Eastern Cooperative Oncology Group Performance Status (ECOG PS) (0-1 vs. ≥ 2), number of metastatic sites (0-1 vs. ≥ 2), use of statins (yes vs. no), history of CV diseases (yes vs. no), DM (yes vs. no), HT (yes vs. no), and BMI (< 25 vs. ≥ 25 kg/m^2^). To estimate the independent prognostic value, multivariable analysis was also performed by using variables with a *P*-value < .05 at univariable analysis. Variables that impact on the circulating lipid profile, such as BMI, statin use at baseline, and sex, were also included in the multivariable model regardless of their significance at univariable. R 3.6.3 (R Project for Statistical Computing) was used for statistical analysis, with all estimates being reported with corresponding 95% confidence intervals and a 2-tailed level of significance of *P* < .05.

## Results

### Patient Clinicopathologic Characteristics

A total of 430 patients with advanced solid tumors treated with ICIs, alone or in combination with TKIs or chemotherapy, were enrolled in the study to analyze the impact of distinct components of circulating lipid profile on outcomes. The baseline availability of components of circulating lipid profile is summarized in Supplementary Fig. S1. The median age at the start of immunotherapy treatment was 69 years old (range: 32-92); 288 (67%) patients were men, and 248 (57%) were current/former smokers. Most patients (266 [62%]) were affected by advanced NSCLC, 373 (87%) underwent ICIs as monotherapy, 235 (55%) received ICIs in first-line setting, and 385 (89%) detained a baseline ECOG PS of 0 or 1 (Table 1).

**Table 1.** Patient clinicopathologic and metabolic characteristics in the whole cohort.

Characteristics	Overall population *n* = 430 (%)
Age at ICI start	
Median (range)	69.0 (32.0-92.0)
Sex	
Female	142 (33.0%)
Male	288 (67.0%)
Smoking status	
Current/former	248 (57.0%)
Never	169 (39%)
NA	13 (4.0%)
Tumor type	
NSCLC	266 (62.0%)
RCC	74 (17.0%)
Melanoma	55 (13.0%)
Others^*^	35 (8.0%)
Treatment type	
ICI	373 (87.0%)
ICI plus chemotherapy	27 (6.0%)
ICI plus TKI	30 (7.0%)
Treatment line	
First	235 (55.0%)
≥ second	195 (45.0%)
ECOG PS	
0-1	385 (89.0%)
≥2	45 (10.0%)
Metastatic sites	
0-1	103 (24.0%)
≥2	327 (76.0%)
Diagnosis of DM	
No	346 (81.0%)
Yes	84 (19.0%)
CV disease	
No	293 (68.0%)
Yes	135 (31.0%)
NA	2 (1.0%)
Hypertension	
No	189 (44.0%)
Yes	241 (56.0%)
Statin use	
No	312 (72.0%)
Yes	115 (27.0%)
NA	3 (1.0%)
BMI	
Underweight (BMI < 18.5)	18 (4.2%)
Normal (18.5 ≤ BMI ≤ 24.9)	213 (49.5%)
Overweight (25 ≤ BMI ≤ 29.9)	135 (31.4%)
Obese (BMI ≥ 30)	60 (13.9%)
NA	4 (0.9%)
TC	
<200 mg/dl	295 (69.0%)
≥200 mg/dl	135 (31.0%)
HDL	
< 40 mg/dl (males) or < 50 mg/dl (females)	92 (21.0%)
≥ 40 mg/dl (males) or ≥ 50 mg/dl (females)	167 (39.0%)
NA	171 (40.0%)
LDL	
< 100 mg/dl	150 (35.0%)
≥ 100 mg/dl	107 (25.0%)
NA	173 (40.0%)
TG	
<150 mg/dl	220 (52.0%)
≥150 mg/dl	96 (22.0%)
NA	114 (26.0%)

^*^Others: urothelial carcinoma, head and neck carcinoma, small cell lung cancer, and breast cancer

Abbreviations: HDL: high-density cholesterol; LDL: low-density cholesterol; ICI: immune checkpoint inhibitor; NSCLC: non-small cell lung cancer; RCC: renal cell carcinoma; TKI: tyrosine kinase inhibitor; ECOG PS: Eastern Cooperative Oncology Group-Performance Status; DM: diabetes mellitus; BMI: body mass index (kg/m^2^); NA: not available; TC: total cholesterol; TG: triglycerides.

### Baseline Lipidic Assessment

Regarding the baseline circulating lipid profile, TC ≥ 200 mg/dl was detected in 135 (31%) patients, HDL < 40 mg/dl for men, and < 50 mg/dl for women in 92 (21%) patients, LDL ≥ 100 mg/dl in 107 (25%) patients, TG ≥ 150 mg/dl in 96 (22%) patients. Patients with TC ≥ 200 mg/dl showed higher concentration of HDL compared to those patients with TC < 200 mg/dl (*P* < .001), higher concentration of LDL (*P* < .001), higher concentration of TG (*P* = .004; (Supplementary Table S1). As expected, patients assuming statins at baseline detained lower plasmatic levels of TC (median TC 156 mg/dl vs. 185 mg/dl, *P* < .001), lower LDL values (median LDL 74 mg/dl vs. 98 mg/dl, *P* < .001); no differences were observed regarding plasma HDL concentration (48 mg/dl vs. 48 mg/dl, *P* = .43). Considering baseline TG, patients with TG ≥ 150 mg/dl showed higher TC levels compared to those with TG < 150 mg/dl (*P* < .001), higher LDL (*P* = .03), lower levels of HDL (*P* = .006; Supplementary Table S2). Baseline lipid profile according to BMI and sex are showed in Supplementary Tables 3 and 4.

### Association Between Circulating Lipid Profile Parameters and Clinical Outcomes

The median follow-up was 32.9 months (95% CI, 27.5-37.4). To address the question regarding the survival impact of plasmatic components of lipid profile, we correlated each variable with PFS, and OS (Supplementary Tables S5 and S6).

#### Total Cholesterol

Considering DCR, no significant differences were observed between patients with TC ≥ 200 mg/dl and < 200 mg/dl (DCR: 60% vs. 56%, *P* = .58). Patients with TC ≥ 200 mg showed a numerically but not significantly longer PFS (median PFS 6.6 vs. 4.7 months, hazard ratio [HR] 0.90, 95% CI, 0.71-1.15, *P* = .4) and a significantly longer OS compared to those with TC < 200 mg/dl (median OS 19.4 vs. 10.8 months, HR 0.73, 95% CI, 0.56-0.95, *P* = .02).

#### Triglycerides

Considering DCR, no significant differences were observed in patients with TG ≥ 150 mg/dl vs. TG < 150 mg/dl (47% vs. 55%, respectively, *P* = .9). Patients with TG ≥ 150 mg/dl showed significantly shorter PFS (median 3.4 vs. 5.1 months, HR 1.39, 95% CI, 1.06-1.82, *P* = .02) and OS (median 7.1 vs. 12.9 months, HR 1.44, 95% CI, 1.09-1.91, *P* = .009) compared to those with TG < 150 mg/dl.

#### LIPID Score

**Figure 1.**
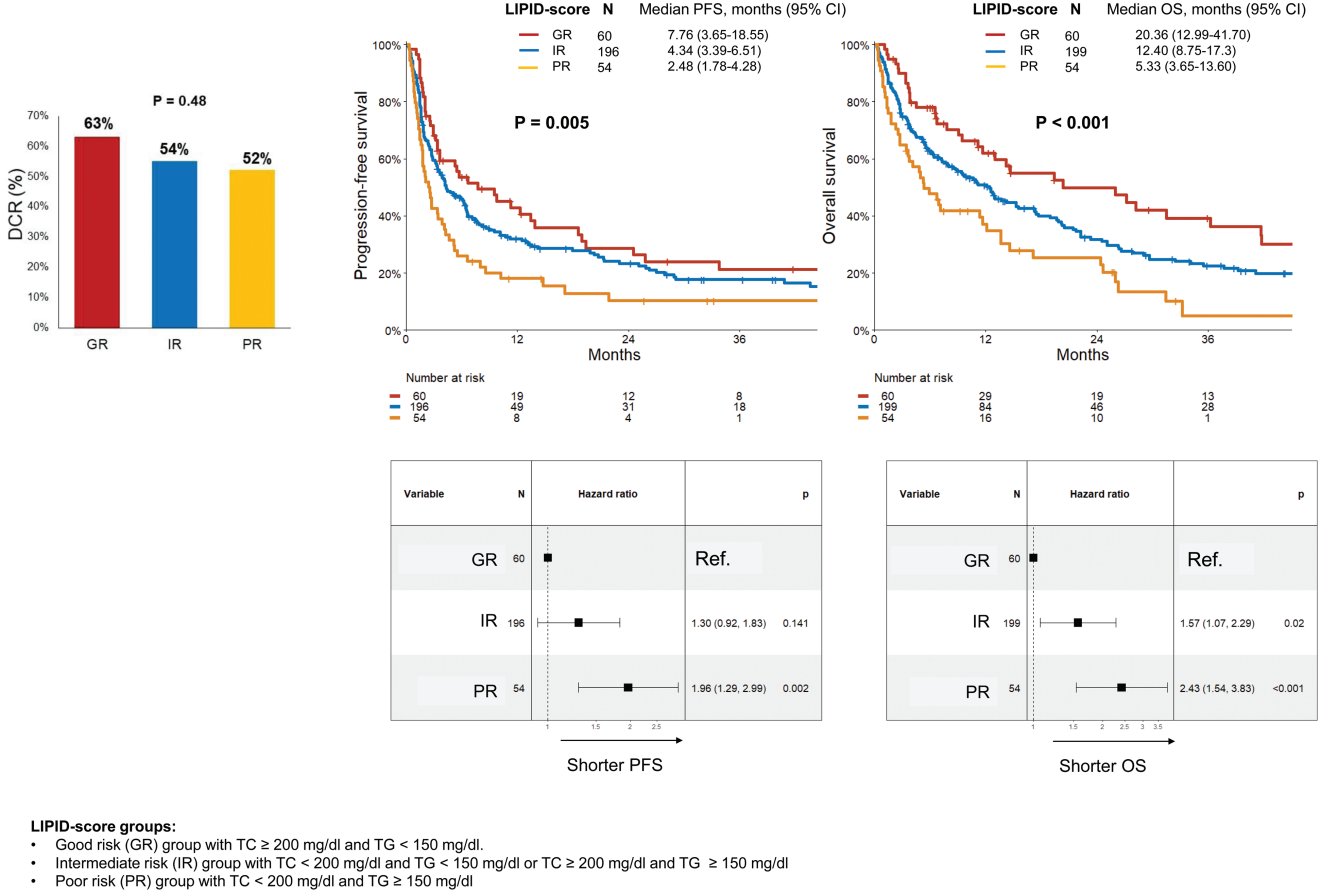
DCR, PFS, and OS according to LIPID score in patients treated with ICI (as monotherapy or combination with chemotherapy or target therapy). Abbreviations: DCR: disease control rate; PFS: progression-free survival; OS: overall survival; LIPID score: good risk (GR) group with TC ≥ 200 mg/dl and TG < 150 mg/dl, intermediate risk (IR) group with TC < 200 mg/dl and TG < 150 mg/dl or TC ≥ 200 mg/dl and TG ≥ 150 mg/dl, poor risk (PR) group with TC < 200 mg/dl and TG ≥150 mg/dl.

We deepened the role of TC and TG as prognostic circulating biomarkers and proposed a lipid score able to discriminate patients that could benefit longer from ICIs. Hence, based on the univariable analysis performed for PFS and OS, we combined TC and TG into a LIPID score that divided our patients into 3 risk groups: good risk (GR) group with TC ≥ 200 mg/dl and TG < 150 mg/dl (*n* = 60, 19%), intermediate risk (IR) group with TC < 200 mg/dl and TG < 150 mg/dl or TC ≥ 200 mg/dl and TG ≥ 150 mg/dl (*n* = 201, 63%), poor risk (PR) group with TC < 200 mg/dl and TG ≥ 150 mg/dl (*n* = 55, 18%). Looking at patient characteristics according to LIPID score, PR group was enriched of patients with a concomitant diagnosis of DM, HTA, CV comorbidities (Table 2). No significant differences in DCR (63% vs. 54% vs. 52% for GR, IR, and PR, respectively, *P* = .48) were observed according to LIPID score. The median PFS of GR, IR, and PR groups was 7.76, 4.34, and 2.48 months, respectively (IR vs. GR: HR 1.30, 95% CI, 0.92-1.83, *P* = .141; PR vs. GR: HR 1.96, 95% CI, 1.29-2.99, *P* = .002). The median OS for GR, IR, and PR groups was 20.4, 12.4, and 5.3 months (IR vs. GR: HR 1.57, 95% CI, 1.07-2.29, *P* = .02; PR vs. GR: HR 2.43, 95% CI, 1.54-3.83, *P* < .001; (Fig. 1). At multivariable analyses, after adjusting for baseline ECOG PS, tumor type, treatment line, sex, statin use, number of metastatic sites, and BMI, the PR represented an independent prognostic factor for both PFS and OS (for PFS: PR vs. GR, HR 1.82, 95% CI, 1.14-2.90, *P* = .01; for OS: PR vs. GR, HR 2.40, 95% CI, 1.46-3.94, *P* < .001), while the IR only for OS (IR vs. GR, HR 1.52, 95% CI, 1.01-2.28, *P* = .04, Table 3).

**Table 2.** Patient clinicopathological and metabolic characteristics according to LIPID score (*n* = 316).

Characteristics	Good risk group (TC ≥ 200 mg/dl and TG < 150 mg/dl) (*n* = 60)	Intermediate risk group TC < 200 mg/dl and TG < 150 mg/dl or TC ≥ 200 mg/dl and TG ≥ 150 mg/dl (*n* = 201)	Poor risk group TC < 200 mg/dl and TG ≥ 150 mg/dl (*n* = 55)	*P*
Age at ICI start				
Median (range)	66.0 (41.0-81.0)	68.0 (31.0-89.0)	69.0 (38.0-86.0)	.09
Sex				
Female	29 (48.3%)	68 (33.8%)	14 (25.5%)	.03
Male	31 (51.7%)	133 (66.2%)	41 (74.5%)	
Tumor type				
NSCLC	39 (65.0%)	128 (63.7%)	34 (61.8%)	.93
Others^*^	21 (35.0%)	73 (36.3%)	21 (38.2%)	
Treatment type				
ICI	49 (81.7%)	174 (86.6%)	52 (94.5%)	.09
ICI plus chemotherapy	6 (10.0%)	7 (3.5%)	1 (1.8%)	
ICI plus TKI	5 (8.3%)	20 (9.9%)	2 (3.6%)	
Treatment line				
First	35 (58.3%)	105 (52.2%)	30 (54.5%)	.70
≥Second	25 (41.7%)	96 (47.7%)	25 (45.5%)	
ECOG PS				
0-1	59 (98.3%)	178 (88.6%)	42 (76.4%)	.01
≥ 2	1 (1.7%)	23 (11.4%)	13 (23.6%)	
Metastatic sites				
0-1	13 (21.7%)	31 (15.4%)	11 (20.0%)	.45
≥ 2	47 (78.3%)	170 (84.6%)	44 (80.0%)	
Diagnosis of DM				
No	56 (93.3%)	163 (81.1%)	39 (70.9%)	.007
Yes	4 (6.7%)	38 (18.9%)	16 (29.1%)	
CV disease				
No	48 (80.0%)	141 (70.2%)	32 (58.2%)	.02
Yes	11 (18.3%)	60 (29.8%)	23 (41.8%)	
NA	1 (1.7%)	0 (0%)	0 (0%)	
Hypertension				
No	25 (41.7%)	92 (45.8%)	13 (23.6%)	.01
Yes	35 (58.3%)	109 (54.2%)	42 (76.4%)	
Statin use				
No	55 (91.7%)	149 (74.1%)	28 (50.9%)	<.001
Yes	4 (6.7%)	51 (25.4%)	27 (49.1%)	
NA	1 (1.7%)	1 (0.5%)	0 (0%)	
Smoking status				
Current/former smoker	37 (61.7%)	117 (58.2%)	21 (38.2%)	.99
Never	23 (38.3%)	75 (37.3%)	33 (60.0%)	
NA	0 (0%)	9 (4.5%)	1 (1.8%)	
BMI (kg/m^2^)				
< 25				
Underweight (BMI < 18.5)	2 (3.3%)	6 (3.0%)	2 (3.6%)	.31
Normal (18.5 ≤ BMI ≤ 24.9)	30 (50.0%)	108 (53.7%)	23 (41.8%)	
≥ 25				
Overweight (25 ≤ BMI ≤ 29.9)	10 (16.7%)	28 (13.9%)	8 (14.5%)	
Obese (BMI ≥ 30)	15 (25.0%)	58 (28.8%)	22 (40.0%)	
NA	3 (5.0%)	1 (0.5%)	0 (0%)	
BMI (kg/m^2^)				
Median (range)	24.1 (16.4-34.3)	24.2 (13.5-43.2)	25.5 (14.7-39.8)	.13
HDL				
Median (range)	63 (38-112)	50 (12-87)	40 (23-73)	<.001
LDL				
Median (range)	137 (78-271)	92 (22-219)	86 (26-133)	<.001

^*^Others: Renal cell carcinoma, melanoma, urothelial carcinoma, head and neck carcinoma, small cell lung cancer, and breast cancer.

Abbreviations: ICI: immune checkpoint inhibitor; NSCLC: non-small cell lung cancer; TKI: tyrosine kinase inhibitor; ECOG PS: Performance Status according to Eastern Cooperative Oncology Group; DM: diabetes mellitus; BMI: body mass index (kg/m^2^); CV: cardiovascular, NA: not available.

**Table 3.** Multivariable analyses for progression-free survival and overall survival in patients treated with ICI (as monotherapy or in combination).

Multivariable analysis						
Test variables	PFS			OS		
	HR	95% CI	*P*	HR	95% CI	*P*
Tumor type						
NSCLC (ref.)/others^*^	0.60	0.44-0.80	<.001	0.58	0.42-0.80	<.001
Treatment lines						
1^st^(ref.)/ ≥ 2	1.44	1.10-1.89	.007	1.46	1.10-1.93	.009
ECOG PS						
0-1 (ref.)/ ≥ 2	2.30	1.53-3.46	<.001	2.30	1.53-3.45	<.001
Metastatic sites						
0-1 (ref.)/ ≥ 2	1.55	1.05-2.27	.02	1.75	1.15-2.68	.01
Sex						
Female (ref.)/ Male	1.11	0.84-1.47	.46	1.40	1.03-1.89	.03
BMI						
< 25 (ref.)/ ≥ 25	0.94	0.71-1.23	.63	0.77	0.58-1.02	.07
Statin use						
no (ref.)/ yes	1.00	0.74-1.35	.99	0.88	0.64-1.20	.41
LIPID score						
GR (ref.)						
IR	1.26	0.87-1.83	.21	1.52	1.01-2.28	.04
PR	1.82	1.14-2.90	.01	2.40	1.46-3.94	<.001

All variables referred to baseline characteristics of patients before ICIs start.

^*^Others: renal cell carcinoma, melanoma, urothelial carcinoma, head and neck cancer, small cell carcinoma, and breast cancer.

Abbreviations: PFS: progression-free survival; OS: overall survival; HR: hazard ratio; CI: confidence interval; NSCLC: non-small cell lung cancer; ECOG PS: Eastern Cooperative Oncology Group Performance Status; BMI: body mass index (kg/m^2^). LIPID score groups: good risk (GR) group with TC ≥ 200 mg/dl and TG < 150 mg/dl; intermediate risk (IR) group with TC < 200 mg/dl and TG < 150 mg/dl or TC ≥200 mg/dl and TG ≥ 150 mg/dl; poor risk (PR) group with TC < 200 mg/dl and TG ≥ 150 mg/dl.

#### TG-HDL Ratio

As in our cohort patients with TC ≥ 200 mg/dl had also higher HDL levels (Supplementary Table S1), and HDL differently from LDL were able to predict OS (see Supplementary Tables S5 and S6 for univariable analysis for LDL and HDL), we combined HDL with TG to investigate the impact of TG-HDL ratio, a marker of insulin resistance and CV risk, on patient prognosis following ICIs treatment^20,21^ in a subgroup of patients with those parameter available at baseline (*N* = 177). The percentage of comorbidities (CV diseases, DM, HT) and statin use at baseline of patients with HDL and LDL available did not differ from those without (Supplementary Tables S7 and S8). TG-HDL ratio was categorized into tertiles, stratifying patients in 3 risk subgroups, as follow: T1 (TG-HDL ratio < 1.76), T2 (1.76 ≤ TG-HDL ratio < 2.92), T3 (≥ 2.92). TG-HDL ratio did not have an impact on DCR (61%, 57%, and 48% for T1, T2, and T3, respectively, *P* = .45). Patients in the T3 subgroup, with higher TG and lower HDL levels, detained shorter PFS compared to those in the T2 and T1 with a median PFS: 7.1 months, 4.3 months, and 2.5 months for T1, T2, T3, respectively (T2 vs. T1, HR 1.25, 95% CI, 0.82-1.92, *P* = .304; T3 vs. T1, HR 1.81, 95% CI, 1.20-2.75, *P* = .005). Moreover, we observed a shorter OS according to higher TG-HDL ratio, with a median OS of 17.4 months for T1, 12.9 months for T2, and 7.5 months for T3, respectively (T2 vs. T1, HR 1.27, 95% CI, 0.80-2.00, *P* = .31; T3 vs. T1, HR 1.73, 95% CI, 1.12-2.69, *P* = .01; Supplementary Fig. S2).

### Impact of LIPID Score and TG-HDL Ratio on Patients Treated With Immunotherapy Alone

To avoid potential effects of chemotherapy and target therapy combined with ICI on patients’ outcomes according to lipid components, we further investigated the impact of LIPID score in the population of patients treated with ICI as monotherapy (*n* = 373). Lipid score was available for 74% of them (*n* = 275). At univariate analysis, PR group was confirmed to detain the worse PFS, and OS compared to the others. The median PFS for GR, IR, and PR was 5.5 months, 3.7 months, and 2.3 months, respectively (IR vs. GR: HR 1.26, 95% CI, 0.89-1.80, *P* = .197; PR vs. GR: HR 1.78, 95% CI, 1.16-2.73, *P* = .008). The median OS for GR, IR, and PR was 19.4 months, 12.3 months, and 5.3 months, respectively (IR vs. GR: HR 1.48, 95% CI, 1.01-2.16, *P* = .04; PR vs. GR: HR 2.22, 95% CI, 1.41-3.50, *P* < .001) (Supplementary Fig. S3); these findings were confirmed at multivariable analysis for both PFS and OS (Supplementary Table S9). We also analyzed the impact of TG-HDL ratio in patients treated with ICI alone (baseline TG-HDL ratio available for 148 patients). We confirmed the negative prognostic impact of high TG-HDL ratio on survival, with patients in the third tertile showing shorter PFS (2.1 months compared to 4.2 months for T1 and 3.6 for T2) and OS (6.9 months compared to 13.2 months for T1 and 12.1 for T2) (for PFS: T2 vs. T1, HR 1.25, 95% CI, 0.81-1.94, *P* = .31, T3 vs.T1, HR 1.68, 95% CI, 1.10-2.59, *P* = .02; for OS: T2 vs. T1, HR 1.25, 95% CI, 0.79-1.98, *P* = .33, T3 vs. T1, HR 1.61, 95% CI, 1.03-2.50, *P* = .04; Supplementary Fig. S4).

## Discussion

In the present study, we observed that the impact of circulating lipid profile on outcome of patients with cancer treated with ICIs may vary according to the parameter considered. In particular, patients with TC ≥ 200 mg/dl showed longer OS compared to those with TC < 200 mg/dl; similarly, patients with higher HDL presented improved OS compared to those with lower plasmatic concentration. Conversely, patients with TG ≥ 150 mg/dl detained significant shorter PFS, and OS compared to those with TG < 150 mg/dl. Interestingly, when we combined TC and TG in the LIPID score, we identified 3 subgroups of patients with distinct survival outcome and the combination of TC < 200 mg/dl and TG ≥ 150 mg/dl revealed the highest negative prognostic value defining a subgroup with worse survival outcome under ICIs treatment. As HDL but not LDL showed an impact on patient outcomes, we also combined HDL with TG in the TG-HDL ratio, a strong indicator of CV risk and insulin resistance. The higher the ratio (higher TG and lower HDL concentrations), the higher the risk of cardiovascular events and insulin resistance and metabolic syndrome. Moreover, it seems to detain a prognostic value in cancer setting.^22^ Our finding identified a subgroup of patients in the third tertile characterized by higher TG levels and lower HDL levels showing a dismal prognosis under ICIs treatment. Patients in the third tertile had a TG-HDL ratio ≥ 2.92, a value similar to the cutoff individuated in other 2 studies for discriminating patients with higher cardiometabolic risk.^23,24^

These results confirm the association between lipid profile parameters, such as TC, TG, and HDL, tumor progression, and tumor immune surveillance. In a cohort of patients with advanced solid cancer treated with ICIs, Perrone et al showed that those with TC ≥ 200 mg/dl had longer OS compared to those with lower TC plasmatic levels.^25^ In line with our results, a study investigating the impact of lipid profile in patients with advanced NSCLC treated with nivolumab found that patients with higher circulating levels of TC and HDL detained longer PFS and OS compared to those with lower levels. This was observed in the nivolumab cohort but not in the chemotherapy control cohort, suggesting a predictive role of lipid profile in the setting of ICIs treatment.^26^

Taken together, these results suggest a positive immunomodulatory function of cholesterol, validating also preclinical evidence supporting its role in strengthening antigen presentation and T-cell activation.^8,27^ Interestingly, the positive modulatory role of HDL in patients with cancer treated with ICIs might be partially explained by the interaction of HDL with ABCA1 and ABCG1 transporters, which not only remove cholesterol from cells, but also modulate T-cells activity and reduce circulating oxidative stress, boosting the antitumor immune response.^28-30^ Our group recently showed that baseline statin use was associated with a better clinical activity of PD-1 inhibitors in malignant pleural mesothelioma and patients with NSCLC.^31^ As statins are drugs commonly used in clinical practice to lower plasmatic cholesterol levels, these results seem to point toward a different direction, yet this discrepancy could be partially explained by the pleiotropic effects of statins as immunomodulatory, antioxidant, and antiproliferative agents beyond their lipid-lowering role.^10,32^

Looking at the role of triglycerides in immune cells modulation, several studies suggested the correlation between hypertriglyceridemia, increased c-reactive protein (CRP), and IL-6 concentration, two serum acute phase reactants with immunosuppressive functions.^33-37^ Preclinical evidence also showed that increased levels of triglycerides impair capacity of dendritic cells to process and present tumor-associated antigens, leading to significantly lower ability to stimulate T cells.^38^ Due to the lack of CRP, IL-6, and MCP-1 levels at baseline, we were not able to validate these correlations in our cohort and further translational studies are needed to better clarify the mechanistic insights supporting the role of TG in immune surveillance against cancer cells.

Noteworthy, our identified LIPID score was able to stratify groups patients with specific metabolic characteristics. As showed in Table 2, the PR subgroup was enriched of patients with a diagnosis of DM, HT, and history of CV events, all comorbidities included in the criteria of metabolic syndrome along with HDL and TG, defining them as patients with metabolic dysfunctional. During last years, immunotherapy researchers focused their attention not only on the specific tumor as an isolate entity inside human body but on the patient as an individual within a specific environment, characterized by a specific metabolic profile, diet habits and lifestyle. Recent studies demonstrated that patients with higher BMI responded better to ICIs, strengthening the evidence that body metabolism and adipose tissue might play a key role in shaping the antitumor immune responses.^18^ In our cohort, we did not observe significant correlations between patient BMI and response to ICIs. BMI alone, even if commonly used in clinical practice, is not able to describe comprehensively the complexity of body composition because it does not take into account body fat distribution.^39^ On the contrary, criteria of metabolic syndrome range from body adipose tissue evaluated by waist circumference to lipid profile, from glycemic balance to CV parameters, ensuring a broader picture of patient metabolic profile.^40^ Among metabolic parameters, not only BMI but also chronic hyperglycemia was correlated with outcomes of patients with cancer treated ICIs. Cortellini et al showed that long-term/poorly controlled diabetes may impair ICIs efficacy.^41^

Cancer is an age-related disease that shares risk factors (obesity, smoking habits, sedentary lifestyle, alcohol consumption, unhealthy diet) with other comorbidities such as CV diseases and diabetes^12,42^; therefore, patients with advanced cancer are often affected by other medical comorbidities that might overall impact on clinical outcomes, even under ICIs treatment.^12-14,43,44^ In our cohort, even if DM, HT, CV diseases alone did not correlate with patient outcomes, LIPID score clearly identified those patients in the PR subgroup characterized by multiple comorbidities related to altered systemic metabolic status, including lower HDL levels, but also CV disease, DM, HT. Therefore, an altered lipid profile may suggest not only impaired antitumor immunity and altered systemic inflammatory status but also an increased cardiovascular risk and metabolic impairment that might compromise overall survival of patients with advanced cancer. In a clinical practice scenario, the LIPID score and TG-HDL ratio may support clinicians in the stratification of patients from a comorbidity point-of-view, defining those patients with higher comorbidities burden (CV disease, DM, HT) and subsequent compromise clinical outcomes under ICIs treatment. In support of these findings, recent evidences showed that increased comorbidity burden defined according to the Charlson comorbidity index (CCI) was associated with decreased OS among patients receiving ICIs.^12^

Some limitations of the study must be acknowledged. First, from each patient, we collected data solely related to the plasmatic lipid content, without an assessment of lipid content of tumor tissues; therefore, no correlations between circulating and tumor tissue lipids could be drawn. Second, due to the retrospective nature of the study, complete lipid profile (TC, TG, HDL, LDL) was not available for all the patients and data on the duration of altered hypercholesterolemia and hypertriglyceridemia before ICI treatment start were also missing, preventing us from inferring a time-depending effect of lipid profile on immune cell phenotype and activity. HDL, and therefore, TG-HDL ratio was available only for a small percentage of patients (*n* = 177), and even if the univariable analysis showed a promising prognostic value of this biomarker, a validation in a larger cohort is needed. In addition, the wide percentage of missing data regarding TG-HDL ratio precluded the chance to fit it into a multivariable model. Third, a detailed nutritional assessment of patients at baseline was not available and, therefore, no correlations between nutritional status and lipid profile could be defined. Finally, due to the lack of a control cohort, we were not able to address the predictive role of circulating lipid profile.

## Conclusion

Nevertheless, our results proposed an easy-to-access lipid score able to stratify patient prognosis under ICIs treatment. The prognostic role of LIPID score, as showed in the multivariable analysis, turned out to be independent of statin intake. The LIPID score was also able to characterize patients with cancer from a metabolic point of view, hinting a baseline inflammatory status and comorbidity burden that may impact on outcome under ICIs treatment. Early assessment and prompt correction of patient metabolic status might then boost anti-cancer immune activity and improve ICIs efficacy. Moreover, the early management of patient comorbidities might improve quality of life and the survival rate overall.

Prospective randomized studies encompassing lipid, metabolic and immune biomarkers are awaited, aiming to validate our lipid signature, evaluate its predictive role, and investigate the impact of early pharmacological intervention on lipid profile in patients treated with ICIs. Further analyses are already ongoing, aimed at exploring the interplay between circulating lipid profile, inflammatory cytokine network, body mass composition, and nutritional status in a subgroup of patients of the same population.

## Supplementary Material

oyad273_suppl_Supplementary_Figures_1

oyad273_suppl_Supplementary_Tables_1-2

## Data Availability

Data are available on reasonable request. The datasets used and/or analyzed during this study are available with the corresponding author on reasonable request.
