# Supplementary material for: Prognostic Impact of Blood Lipid Profile in Patients With Advanced Solid Tumors Treated With Immune Checkpoint Inhibitors: A Multicenter Cohort Study
Source: Oncologist. 2023 Oct 5;29(3):e372–81. doi: 10.1093/oncolo/oyad273 (PMC10911919; doi:10.1093/oncolo/oyad273)
Supplement: oyad273_suppl_Supplementary_Figures_1 [file oyad273_suppl_supplementary_figures_1.docx]

**Supplementary Figures**

**Supplementary Figure 1.** Flow diagram of study population according to baseline components of circulating lipid profile availability.

**
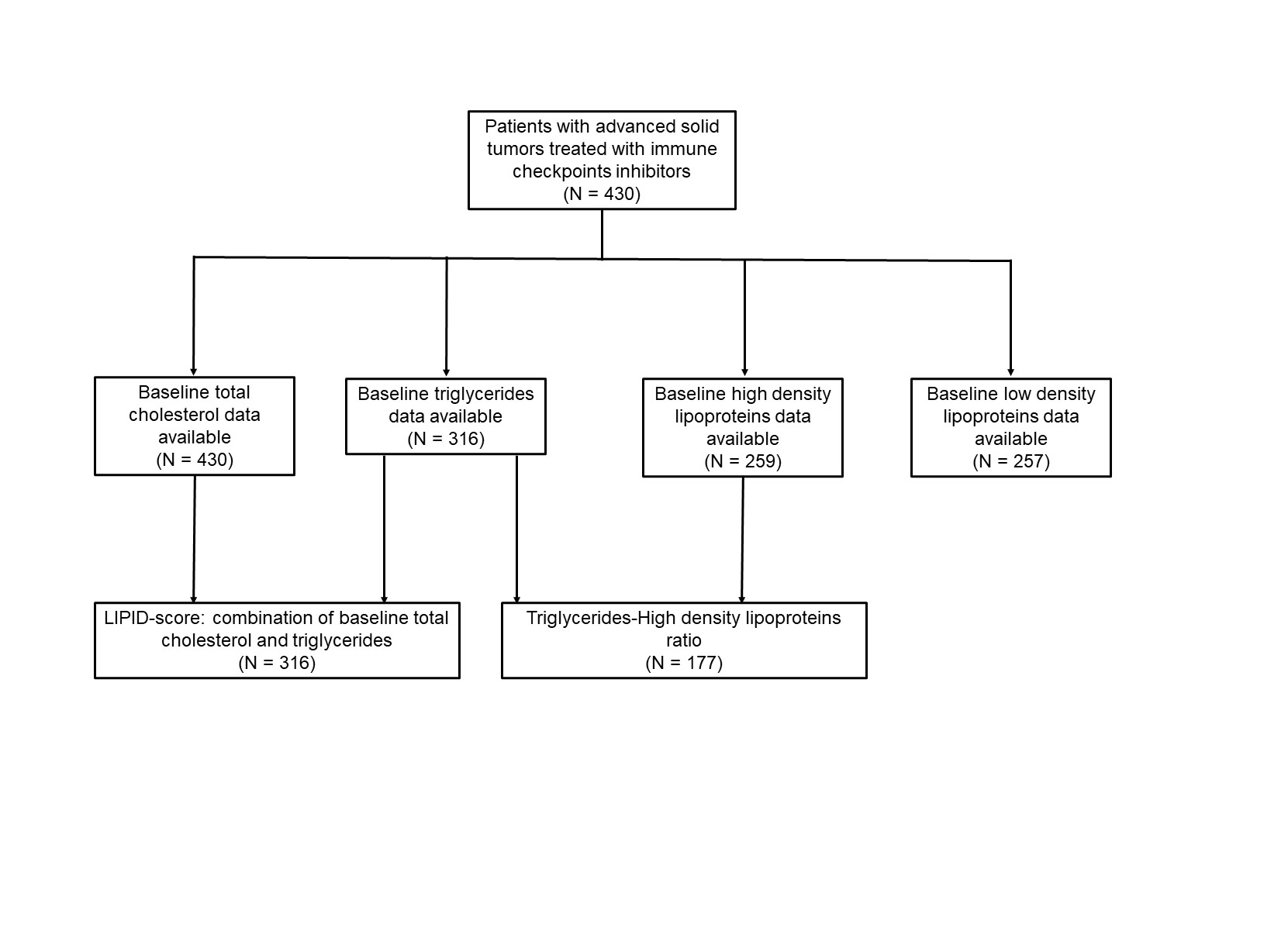
**

**Supplementary Figure 2.** DCR, PFS and OS according to TG-HDL Ratio in patients treated with ICI (as monotherapy or combination with chemotherapy or target therapy).


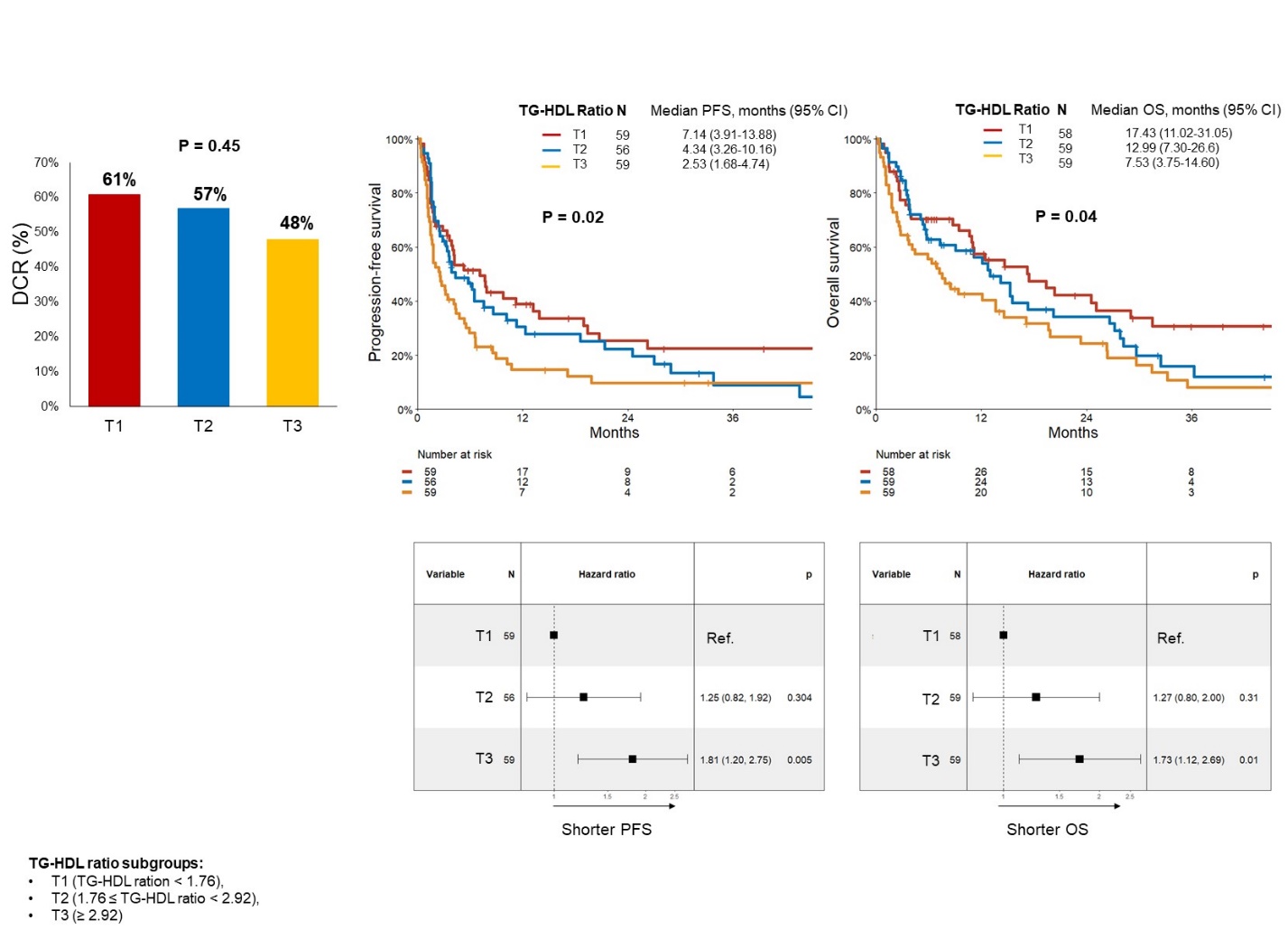


**Abbreviations:** DCR, disease control rate, PFS, progression free survival, OS, overall survival, T1, first tertile subgroup (lower triglycerides levels and higher HDL levels), T2, second tertile subgroup, T3, third tertile subgroup (higher triglycerides levels and lower HDL levels): T1 (TG-HDL ratio < 1.76), T2 (1.76≤ TG-HDL ratio<2.92), T3 (TG-HDL ratio≥ 2.92).

**Supplementary Figure 3.** DCR, PFS and OS according to LIPID-score only in patients treated with ICI as monotherapy


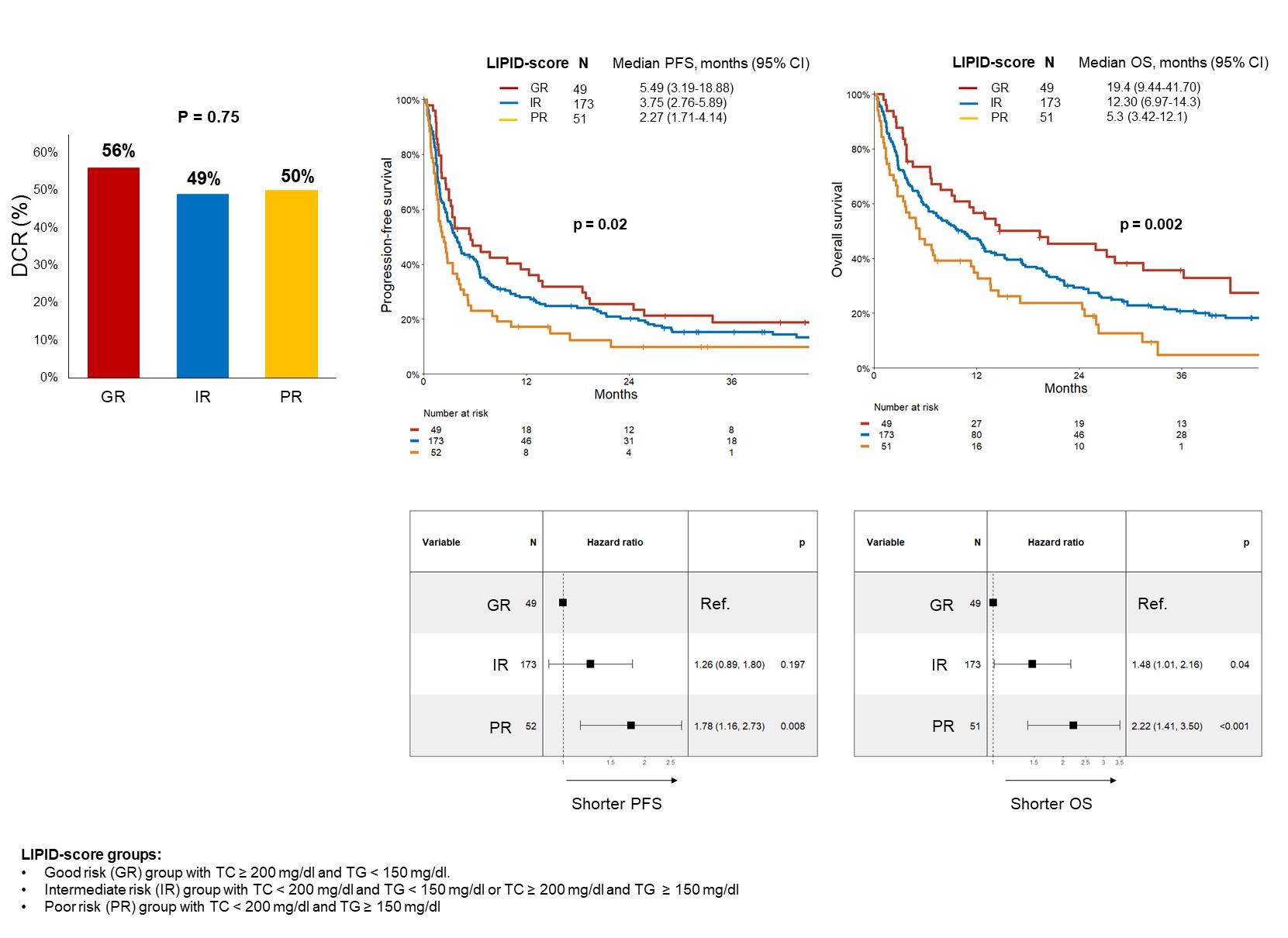


**Abbreviations:** DCR, disease control rate, PFS, progression free survival, OS, overall survival, LIPID-score: good risk (GR) group with TC ≥ 200 mg/dl and TG < 150 mg/dl, intermediate risk (IR) group with TC < 200 mg/dl and TG < 150 mg/dl or TC ≥ 200 mg/dl and TG ≥ 150 mg/dl, poor risk (PR) group with TC < 200 mg/dl and TG ≥ 150 mg/dl.

**Supplementary Figure 4.** DCR, PFS, and OS according to TG-HDL ratio in patients treated with ICI as monotherapy

**
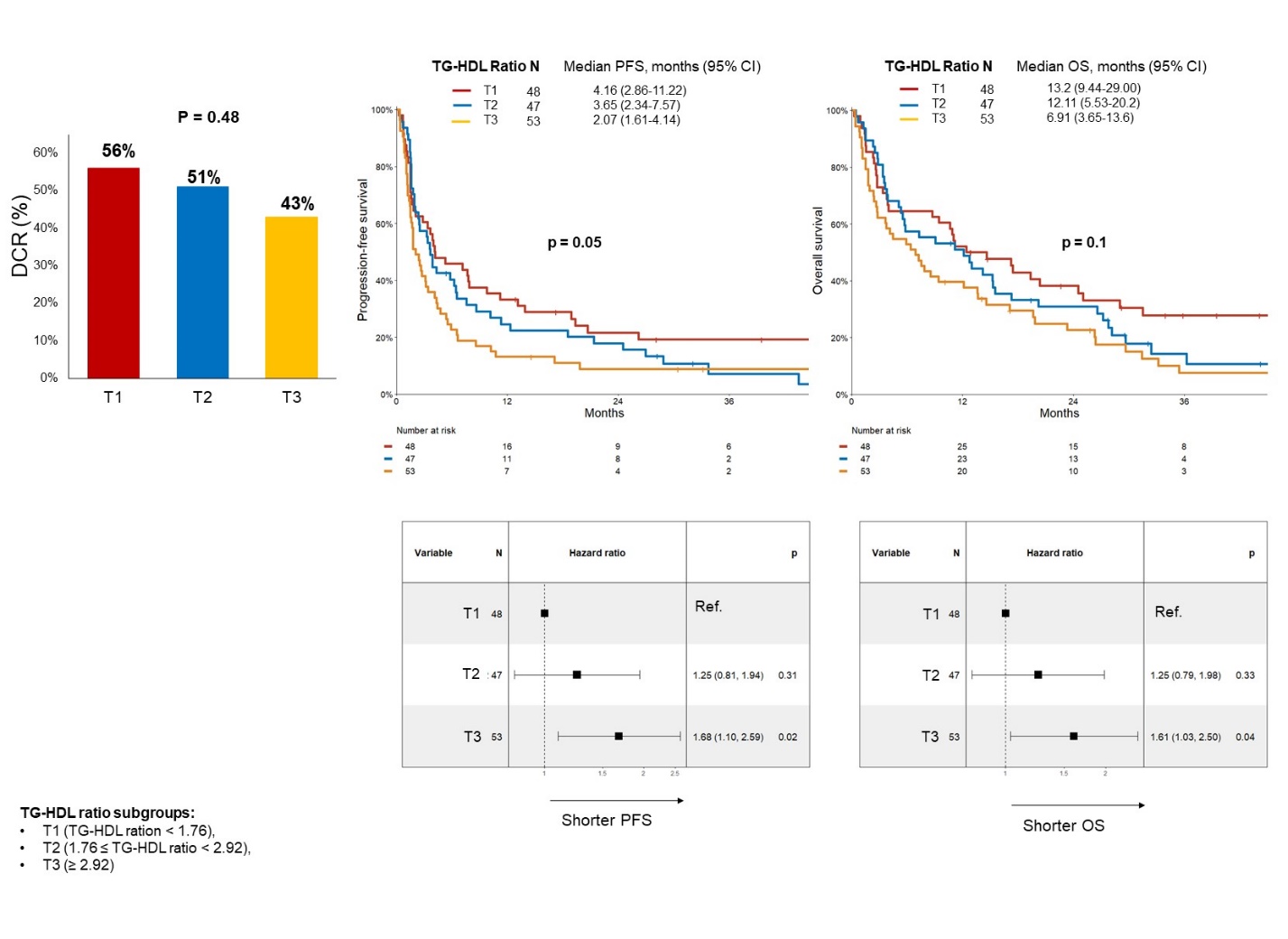
**

**Abbreviations:** DCR, disease control rate, PFS, progression free survival, OS, overall survival, T1, first tertile subgroup (lower triglycerides levels and higher HDL levels), T2, second tertile subgroup, T3, third tertile subgroup (higher triglycerides levels and lower HDL levels): T1 (TG-HDL ration < 1.76), T2 (1.76≤ TG-HDL ratio<2.92), T3 (TG-HDL ratio ≥ 2.92).
