# Supplementary material for: Prognostic Impact of Blood Lipid Profile in Patients With Advanced Solid Tumors Treated With Immune Checkpoint Inhibitors: A Multicenter Cohort Study
Source: Oncologist. 2023 Oct 5;29(3):e372–81. doi: 10.1093/oncolo/oyad273 (PMC10911919; doi:10.1093/oncolo/oyad273)
Supplement: oyad273_suppl_Supplementary_Tables_1-2 [file oyad273_suppl_supplementary_tables_1-2.docx]

**Supplementary Materials**

**Supplementary Tables**

**Supplementary Table 1.** Patient circulating lipid profile and body mass index according to total cholesterol levels.

|  | **Total cholesterol ≥ 200 mg/dl  (N = 135)** | **Total cholesterol < 200 mg/dl (N = 295)** | ***p*** |
| --- | --- | --- | --- |
| **BMI** |  |  |  |
| Median [range] | 24.55 [13.5-34.50] | 24.22 [14.7-42.20] | 0.52 |
| **HDL (mg/dl)** |  |  |  |
| Median [range] | 60.0 [27-112] | 45.0 [11-87] | **< 0.001** |
| **LDL (mg/dl)** |  |  |  |
| Median [range] | 135 [58-271] | 82.5 [22-137] | **< 0.001** |
| **TG (mg/dl)** |  |  |  |
| Median [range] | 126 [48-308] | 106 [47-312] | **0.004** |

Body mass index (BMI), total cholesterol (TC), triglycerides (TG), low-density lipoproteins (LDL), high density lipoproteins (HDL).

**Supplementary Table 2.** Patient circulating lipid profile and body mass index according to triglycerides levels.

|  | **Triglycerides ≥ 150 mg/dl (N = 96)** | **Triglycerides < 150 mg/dl (N = 220)** | ***p*** |
| --- | --- | --- | --- |
| **BMI** |  |  |  |
| Median [range] | 25.5 [13.5-39.8] | 24 [15.6-43.2] | **0.01** |
| **TC (mg/dl)** |  |  |  |
| Median [range] | 192 [92-336] | 173 [59-310] | **< 0.001** |
| **HDL (mg/dl)** |  |  |  |
| Median [range] | 46 [23-86] | 53 [12-112] | **0.006** |
| **LDL (mg/dl)** |  |  |  |
| Median [range] | 111 [26-219] | 96 [22-271] | **0.03** |

Body mass index (BMI), total cholesterol (TC), triglycerides (TG), low-density lipoproteins (LDL), high density lipoproteins (HDL).

**Supplementary Table 3.** Patient circulating lipid profile according to BMI

|  | **BMI <25**  **(n = 231)** | **BMI ≥ 25 (n = 195)** | ***p*** |
| --- | --- | --- | --- |
| **TC (mg/dl)** |  |  |  |
| Median [range] | 175 [59-336] | 178 [89-309] | 0.95 |
| **TG (mg/dl)** |  |  |  |
| Median [range] | 107 [47-308] | 120 [52-312] | 0.06 |
| **HDL (mg/dl)** |  |  |  |
| Median [range] | 49 [11-112] | 47 [21-92] | 0.72 |
| **LDL (mg/dl)** |  |  |  |
| Median [range] | 94 [22-198] | 93.5 [26-219] | 0.88 |

Body mass index (BMI), total cholesterol (TC), triglycerides (TG), low-density lipoproteins (LDL), high density lipoproteins (HDL).

**Supplementary Table 4.** Patient circulating lipid profile and body mass index according to sex.

|  | **Female (n = 142)** | **Male (n = 288 )** | ***p*** |
| --- | --- | --- | --- |
| **BMI** |  |  |  |
| Median [range] | 23.0 [13.5-39.4] | 25.0 [16.1-43.2] | **0.001** |
| **TC (mg/dl)** |  |  |  |
| Median [range] | 193 [82-310] | 171 [59-336] | **< 0.001** |
| **HDL (mg/dl)** |  |  |  |
| Median [range] | 54 [26-112] | 45 [11-91.0] | **< 0.001** |
| **LDL (mg/dl)** |  |  |  |
| Median [range] | 96 [30-192] | 89.5 [22-271] | **0.03** |
| **TG (mg/dl)** |  |  |  |
| Median [range] | 104 [48-308] | 120 [47-312] | **0.05** |

Body mass index (BMI), total cholesterol (TC), triglycerides (TG), low-density lipoproteins (LDL), high density lipoproteins (HDL).

**Supplementary Table 5.** Univariable analysis for progression-free survival in patients treated with ICI (as monotherapy or combination with chemotherapy or target therapy).

| **Variables** | **N*** | **mPFS (months)** | **HR [95% CI]** | ***p* value** |
| --- | --- | --- | --- | --- |
| Age (< 70-ref vs ≥ 70 yo) | 209 vs 210 | 6.22 vs 4.74 | 1.22 [0.97-1.52] | 0.08 |
| Sex (Female-ref vs Male) | 140 vs 279 | 6.55 vs 5.13 | 1.11 [0.88-1.41] | 0.4 |
| Tumor type (NSCLC-ref vs Others******) | 263 vs 156 | 4.28 vs 6.94 | 0.60 [0.47-0.77] | **< 0.001** |
| Treatment lines (1st-ref vs ≥ 2nd) | 226 vs 193 | 8.59 vs 3.65 | 1.64 [1.31-2.05] | **< 0.001** |
| ECOG-PS (0-1 ref vs ≥2) | 376 vs 43 | 6.25 vs 1.53 | 2.53 [1.79-3.56] | **< 0.001** |
| Metastatic sites (< 2-ref vs ≥ 2) | 98 vs 321 | 11.32 vs 4.34 | 1.74 [1.31-2.31] | **< 0.001** |
| Baseline statin use (no-ref vs yes) | 305 vs 111 | 5.49 vs 5.46 | 1.16 [0.91-1.48] | 0.5 |
| DM (no-ref vs yes) | 336 vs 83 | 5.72 vs 4.44 | 1.18 [0.90-1.55] | 0.2 |
| HT (no-ref vs yes) | 182 vs 237 | 6.55 vs 4.67 | 1.18 [0.94-1.48] | 0.1 |
| CV diseases in anamnesis (no-ref vs yes) | 283 vs 134 | 5.89 vs 5.13 | 1.12 [0.88-1.42] | 0.3 |
| BMI (< 25-ref vs ≥ 25) | 224 vs 191 | 5.89 vs 5.72 | 0.89 [0.71-1.12] | 0.6 |
| BMI (< 25-ref vs ≥ 25)******* | 207 vs 190 | 6.02 vs 5.72 | 1.05 [0.75-1.20] | 0.7 |
| TC (<200 mg/dl-ref vs ≥ 200 mg/dl) | 286 vs 133 | 4.67 vs 6.61 | 0.90 [0.71-1.15] | 0.4 |
| HDL (≥ 40 mg/dl for M and ≥ 50 mg/dl for F-ref vs < 40 mg/dl for M and < 50 mg/dl for F] | 162 vs 91 | 5.7 vs 4.4 | 1.10 [0.82-1.49] | 0.5 |
| LDL (< 100 mg/dl-ref vs ≥ 100 mg/dl) | 147 vs 106 | 4.34 vs 5.79 | 1.04 [0.78-1.39] | 0.8 |
| TG (< 150 mg/dl-ref vs ≥ 150 mg/dl) | 215 vs 95 | 5.07 vs 3.42 | 1.39 [1.06-1.82] | **0.02** |

**Abbreviations:** n, number; pts, patients; mo, months; vs; versus; mPFS, median progression free survival, yo, years-old; NSCLC, non-small cell lung cancer; ECOG-PS, Eastern Cooperative Oncology Group-Performance Status; DM, diabetes mellitus; HT, hypertension, CV, cardiovascular BMI, body mass index; TC, total cholesterol; TG, triglycerides, HDL, high density cholesterol; LDL, low density cholesterol.

*****Number of patients with data available for PFS analysis

******Others: Renal cell carcinoma, melanoma, urothelial carcinoma, head and neck carcinoma, small cell lung cancer, breast cancer.

*******After removing underweighted patients (n=18 patients).

**Supplementary Table 6.** Univariable analysis for overall survival in patients treated with ICI (as monotherapy or combination with chemotherapy or target therapy).

| **Variables** | **n*** | **mOS (months)** | **HR (95% CI)** | ***p* value** |
| --- | --- | --- | --- | --- |
| Age (< 70-ref vs ≥ 70 yo) | 213 vs 214 | 14.6 vs 10.9 | 1.23 [0.97-1.56] | 0.07 |
| Sex (Female-ref vs Male) | 142 vs 285 | 19.7 vs 10.6 | 1.36 [1.06-1.7] | **0.02** |
| Tumor type (NSCLC-ref vs Others**) | 265 vs 162 | 10.3 vs 21.4 | 0.60 [0.47-0.80] | **< 0.001** |
| Treatment lines (1st-ref vs ≥2nd) | 233 vs 196 | 19.8 vs 7.86 | 1.63 [1.29-2.06] | **< 0.001** |
| ECOG-PS (0-1 ref vs ≥2) | 382 vs 45 | 14.5 vs 2.57 | 3.41 [2.42-4.79] | **< 0.001** |
| Metastatic sites (<2-ref vs ≥ 2) | 102 vs 325 | 27.2 vs 10.1 | 2.1 [1.53-2.87] | **< 0.001** |
| Baseline statin use (no-ref vs yes) | 309 vs 115 | 12.5 vs 12.9 | 1.09 [0.84-1.41] | 0.5 |
| DM (no-ref vs yes) | 343 vs 84 | 12.9 vs 11.3 | 1.17 [0.88-1.56] | 0.3 |
| HT (no-ref vs yes) | 186 vs 241 | 17.11 vs 11.3 | 1.25 [0.98-1.58] | 0.07 |
| CV diseases in anamnesis (no-ref vs yes) | 290 vs 135 | 13.7 vs 10.3 | 1.19 [0.93-1.53] | 0.1 |
| BMI (< 25-ref vs ≥ 25) | 228 vs 195 | 11.7 vs 14.6 | 0.82 [0.65-1.05] | 0.1 |
| BMI (< 25-ref vs ≥25)*** | 211 vs 194 | 11.7 vs 14.6 | 0.83 [0.65-1.05] | 0.1 |
| TC (< 200 mg/dl-ref vs ≥ 200 mg/dl) | 292 vs 135 | 10.8 vs 19.4 | 0.73 [0.56-0.95] | **0.02** |
| HDL (≥ 40 mg/dl for M and ≥ 50 mg/dl for F-ref vs < 40 mg/dl for M and < 50 mg/dl for F] | 167 vs 91 | 17.3 vs 10.3 | 1.49 [1.09-2.04] | **0.01** |
| LDL (< 100 mg/dl-ref vs ≥ 100 mg/dl) | 150 vs 106 | 10.6 vs 14.6 | 0.88 [0.64-1.20] | 0.4 |
| TG (< 150 mg/dl-ref vs ≥ 150 mg/dl) | 218 vs 95 | 12.93 vs 7.14 | 1.44 [1.09-1.91] | **0.009** |

**Abbreviations:** n, number; pts, patients; mo, months; vs; versus; mOS, median overall survival, yo, years-old; NSCLC, non-small cell lung cancer; ECOG PS, Eastern Cooperative Oncology Group-Performance Status; DM, diabetes mellitus; HT, hypertension, CV, cardiovascular, BMI, body mass index; TC, total cholesterol; TG, triglycerides, HDL, high density cholesterol; LDL, low density cholesterol.

*****Number of patients with data available for PFS analysis

******Others: Renal cell carcinoma, melanoma, urothelial carcinoma, head and neck carcinoma, small cell lung cancer, breast cancer.

*******After removing underweighted patients (n=18 patients)

**Supplementary Table 7.** Distribution of comorbidities between patients with available LDL values at baseline and those without.

|  | **LDL not available**  **(N=173)** | **LDL available**  **(N=257)** | **P** |
| --- | --- | --- | --- |
| **CV** |  |  |  |
| No | 125 (72.7%) | 168 (65.6%) | 0.15 |
| Yes | 47 (27.3%) | 88 (34.4%) |  |
| NA | 1 | 1 |  |
| **DM** |  |  |  |
| No | 135 (78.0%) | 211 (82.1%) | 0.36 |
| Yes | 38 (22.0%) | 46 (17.9%) |  |
| **HT** |  |  |  |
| No | 75 (43.4%) | 114 (44.4%) | 0.91 |
| Yes | 98 (56.6%) | 143 (55.6%) |  |
| **Baseline statin use** |  |  |  |
| No | 116 (68.2%) | 196 (76.3%) | 0.09 |
| Yes | 54 (31.8%) | 61 (23.7%) |  |
| NA | 3 | 0 |  |

Abbreviations: CV, cardiovascular; DM, diabetes mellitus; HT, hypertension; NA: not available

**Supplementary Table 8.** Distribution of comorbidities between patients with available HDL values at baseline and those without.

|  | **HDL not available**  **(N=171)** | **HDL available**  **(N=259)** | **P** |
| --- | --- | --- | --- |
| **CV disease** |  |  |  |
| No | 121 (71.2%) | 172 (66.7%) | 0.38 |
| Yes | 49 (28.8%) | 86 (33.3%) |  |
| NA | 1 | 1 |  |
| **DM** |  |  |  |
| No | 136 (79.5%) | 210 (81.1%) | 0.78 |
| Yes | 35 (20.5%) | 49 (18.9%) |  |
| **HT** |  |  |  |
| No | 74 (43.3%) | 115 (44.4%) | 0.89 |
| Yes | 97 (56.7%) | 144 (55.6%) |  |
| **Baseline statin use** |  |  |  |
| No | 115 (68.5%) | 197 (76.1%) | 0.10 |
| Yes | 53 (31.5%) | 62 (23.9%) |  |
| NA | 3 | 0 |  |

Abbreviations: CV, cardiovascular; DM, diabetes mellitus; HT, hypertension; NA: not available

| **Supplementary Table 9.** Multivariable analyses for progression-free survival and overall survival in patients treated with ICI as monotherapy **Multivariable analysis** | | | | | | |
| --- | --- | --- | --- | --- | --- | --- |
|  | **PFS** | | | **OS** | | |
| **Test variables** | **HR** | **95% CI** | ***p*** | **HR** | **95% CI** | ***p*** |
| **Tumor type** | | | | | | |
| NSCLC (ref.) / Others* | 0.62 | 0.46-0.84 | **0.002** | 0.60 | 0.44-0.83 | **0.002** |
| **Treatment lines** | | | | | | |
| 1^st^(ref.) / ≥ 2 | 1.51 | 1.15-2.00 | **0.003** | 1.49 | 1.12-2.0 | **0.007** |
| **ECOG PS** | | | | | | |
| 0-1 (ref.) / ≥ 2 | 2.60 | 1.72-3.94 | **< 0.001** | 2.53 | 1.67-3.83 | **< 0.001** |
| **Metastatic sites** | | | | | | |
| 0-1 (ref.) / ≥ 2 | 1.58 | 1.06-2.35 | **0.023** | 1.75 | 1.14-2.70 | **0.01** |
| **Sex** | | | | | | |
| Female (ref.) / Male | 1.09 | 0.82-1.46 | 0.54 | 1.39 | 1.02-1.91 | **0.04** |
| **BMI** | | | | | | |
| < 25 (ref.)/ ≥ 25 | 0.96 | 0.73-1.27 | 0.78 | 0.78 | 0.58-1.04 | 0.09 |
| **Statin use** | | | | | | |
| no (ref.) / yes | 1.05 | 0.77-1.43 | 0.76 | 0.93 | 0.67-1.28 | 0.64 |
| **LIPID-score** | | | | | | |
| GR (ref.) |  |  |  |  |  |  |
| IR | 1.24 | 0.85-1.81 | 0.21 | 1.47 | 0.97-2.22 | 0.07 |
| PR | 1.91 | 1.19-3.07 | **0.007** | 2.45 | 1.49-4.05 | **< 0.001** |

**Abbreviations:** PFS, progression free survival; OS, overall survival; HR, hazard ratio; CI, confidence interval; NSCLC, non-small cell lung cancer; ECOG PS; Eastern Cooperative Oncology Group Performance Status; BMI, body mass index (Kg/m^2^).**LIPID-score groups:** good risk (GR) group with TC ≥ 200 mg/dl and TG < 150 mg/dl; intermediate risk (IR) group with TC < 200 mg/dl and TG < 150 mg/dl or TC ≥ 200 mg/dl and TG ≥ 150 mg/dl; poor risk (PR) group with TC < 200 mg/dl and TG ≥ 150 mg/dl. All variables referred to baseline characteristics of patients before ICIs start.

*Others: renal cell carcinoma, melanoma, urothelial carcinoma, head and neck cancer, small cell carcinoma, breast cancer.
